# Lesion-Induced Changes to the Network Controllability of the Right Pars Triangularis in Aphasia

**DOI:** 10.1162/nol.a.11

**Published:** 2025-09-02

**Authors:** Harrison Stoll, Apoorva Kelkar, Peter E. Turkeltaub, Roy H. Hamilton, Branch Coslett, John D. Medaglia

**Affiliations:** Department of Psychological and Brain Sciences, Drexel University, Philadelphia, PA, USA; Department of Neurology, Georgetown University, Washington, DC, USA; MedStar National Rehabilitation Hospital, Washington, DC, USA; Department of Neurology, University of Pennsylvania, Hershey, PA, USA; Department of Neurology, Drexel University, Philadelphia, PA, USA

**Keywords:** aphasia, connectome, language, stroke, structure

## Abstract

Left hemisphere stroke causes functional changes to the language network and may shift aspects of language processing to right hemisphere homotopes of perisylvian language regions. The result of right hemisphere recruitment is unclear. Studies suggest the right pars triangularis (rPTr) engagement in language processing corresponds to higher dysfunction. As a result, the region is a site for inhibitory neuromodulation, with evidence that inhibiting the region improves language function in persons with aphasia (PWA). However, studies have also found no relationship between rPTr functional activity and language performance in PWA. The mixed evidence regarding the rPTr suggests additional work is needed to understand the role of the region in PWA. We propose that the white matter connections that support communication between regions may be an important mediator. Thus, we sought to investigate if left hemisphere stroke leads to changes in the structural topological properties of the region. We used measures from network control theory (NCT) to compare the theoretical capacity of the rPTr to integrate communication across brain modules (i.e., boundary controllability [BC]) in the brain, in 60 PWA and 62 controls. We also examined whether BC corresponded to different aspects of language processing (i.e., semantic and phonological) in PWA. We found that PWA had a higher BC in the rPTr relative to controls. Higher BC was associated with fewer phonological errors in a picture naming task. These findings suggest that left hemisphere stroke causes shifts in the structural role of right hemisphere regions that relate to language processing in PWA.

## INTRODUCTION

Aphasia impacts approximately 20%–40% of left hemisphere stroke survivors, and it causes a loss in communication skills that can have a debilitating impact on quality of life (Caporali & Basso, 2003; Ross & Wertz, 2003; Watila & Balarabe, 2015). Despite reports that over 40% of persons with aphasia (PWA) still have significant language deficits 1 year after stroke (Laska et al., 2001), our understanding of aphasia recovery is relatively poor. One of the main outstanding concerns in aphasia research we aim to address is how the right pars triangularis (rPTr) contributes to language recovery in persons with aphasia (PWA).

After a stroke, language network reorganization includes domain-general regions in the left and right hemispheres homotopes of perisylvian language regions (Buckner et al., 1996; Gold & Kertesz, 2000; Rosen et al., 2000; Saur et al., 2006; Stockert et al., 2020; Warburton et al., 1999). Language networks reorganize depending on the amount of remaining tissue in the left hemisphere language network (Brownsett et al., 2014; Skipper-Kallal et al., 2017b; Turkeltaub et al., 2011) and frontoparietal control regions (Geranmayeh et al., 2016, 2017; Heiss et al., 2003; Suárez et al., 2020). Less structural damage to these regions, and higher functional activation, is often associated with better performance in PWA (Griffis et al., 2019; Siegel et al., 2016). Researchers have often demonstrated that the amount of surviving tissue in the left hemisphere perisylvian network is the most important predictor of language recovery in PWA (Hope et al., 2013; Plowman et al., 2012).

Amid severe left hemisphere damage, the right hemisphere becomes important for language network reorganization (Leff et al., 2002; Saur et al., 2006; Skipper-Kallal et al., 2017a, 2017b; Xing et al., 2016). Acute recovery often recruits the right hemisphere, including frontal regions (Saur et al., 2006; Stockert et al., 2020), and larger lesions that include domain-general and perisylvian language regions may require additional support from the right hemisphere in the chronic phases of recovery (Kiran et al., 2015; Saur et al., 2006; Thompson et al., 2010). There is structural and functional evidence that the right temporal lobe plays a supportive role in chronic language recovery (Hope et al., 2017; Skipper-Kallal et al., 2017a; Xing et al., 2016). For example, higher functional activity of temporoparietal regions in the right hemisphere was associated with better language processing in PWA (Skipper-Kallal et al., 2017a). Thus, evidence from some research supports the claim that right hemisphere involvement improves language processing in PWA.

In contrast, right hemisphere involvement in chronic language network reorganization may also correspond to worse language outcomes (Fernandez et al., 2004; Kurland et al., 2008). Specifically, there is mixed evidence about the role of the rPTr and its activity in language recovery. During overt picture naming, rPTr functional activity was higher on error trials than accurate trials (Cao et al., 1999; Postman-Caucheteux et al., 2010). Moreover, inhibitory neuromodulation of the rPTr was often associated with improved overt picture naming performance (Hamilton et al., 2010; Harvey et al., 2019; Naeser, Martin, Nicholas, Baker, Seekins, Helm-Estabrooks, et al., 2005; Naeser, Martin, Nicholas, Baker, Seekins, Kobayashi, et al., 2005). This line of evidence, in conjunction with the finding that the rPTr has high functional activity in PWA (Turkeltaub et al., 2011), led several researchers to claim that the rPTr engaging in language function causes language dysfunction (Barwood et al., 2011; Naeser et al., 2004; Naeser, Martin, Nicholas, Baker, Seekins, Helm-Estabrooks, et al., 2005; Ren et al., 2014). However, recent evidence has also provided evidence against the association between rPtr and language dysfunction. For example, several studies found no relationship between rPTr functional activity and naming task performance in PWA (Skipper-Kallal et al., 2017a, 2017b). Furthermore, the evidence that inhibiting the rPTr with neuromodulation is mixed, with not all patients showing improvements in naming performance (Hamilton et al., 2011; Martin et al., 2009; Turkeltaub et al., 2012). Therefore, the role of the rPTr has remained elusive, and additional perspectives could be needed to understand the role of the region in language recovery for PWA.

In the context of mixed functional evidence, we suggest that an understudied facet of aphasia recovery is the relationship between structural disconnections in the left hemisphere and structural connectivity changes in the right hemisphere. Due to the fact that lesions will typically damage multiple brain regions and connections, they will induce complex effects on brain structural network topology post-aphasia. Furthermore, prior research demonstrated the importance of structural disconnection in understanding functional connectivity and network organization changes that occurred after stroke (Griffis et al., 2019, 2020; Medaglia et al., 2022; Siegel et al., 2016, 2018; Wilmskoetter et al., 2022). In the context of aphasia, structural disconnection predicted language deficits, and the preserved network topological properties of language regions have been related to language function (Erickson et al., 2022; Gleichgerrcht et al., 2016; Griffis et al., 2020; Medaglia et al., 2022; Siegel et al., 2015). Other lines of research used graph theoretical measures to determine how topological properties of language regions in healthy controls (Medaglia et al., 2021; Medaglia, Harvey, et al., 2018) and in PWA (Gleichgerrcht et al., 2016; Gratton et al., 2013; Ketchabaw et al., 2022) related to individual differences in language processing. These findings have demonstrated that higher structural disconnection in the left hemisphere relates to worse aphasia recovery. However, the impact of structural changes in the left hemisphere on the right hemisphere regions has been underexamined. It is possible that shifts in right hemisphere roles mediate shifts in functional roles and behavior.

One approach to understanding how left hemisphere stroke may cause changes to the structural role of right hemisphere regions, specifically the rPTr, is through network control theory (NCT; Ruths & Ruths, 2014). Like graph theory, NCT uses discrete elements of a network (e.g., *nodes*) and the connections between them (e.g., *edges*) to examine the network properties of a region. Unlike traditional graph theory measures, NCT provides a measure of the theoretical ability of a node to drive the network to a desired state, such as integrating or segregating communities (i.e., nodes of a network that are strongly related to one another) to achieve a specific neural state for a context-specific goal. In the context of aphasia, lower controllability of the superior temporal gyrus mediated the relationship between advanced brain age and aphasia severity (Wilmskoetter et al., 2023). However, the role of the rPTr specifically has not been explored, and other forms of controllability maybe relevant for the region. Specifically, the measure *boundary controllability* (BC), the theoretical capacity of a region to integrate or segregate communities of a network, could be related to the cognitive role the rPTr has in language processing. BC represents a measure of interest for understanding the role of right hemisphere regions in language processing, including the rPTr. Non-language-specific regions in the right hemisphere may require increased interaction with the residual language network and supporting domain-general areas to assist language function.

Consequently, we may anticipate that PWA will exhibit higher BC in right hemisphere regions dedicated to language processing compared to healthy controls. Moreover, if the involvement of the region is associated with better language processing, we might expect higher BC to reflect better language outcomes. This is because the right hemisphere region can directly engage (i.e., integrate or segregate) with the left hemisphere via homotopic connections or indirect pathways (Erickson et al., 2022; Griffis et al., 2020). However, if BC is too high in the rPTr, it could place too many demands on the regions, which leads to language dysfunction (Medaglia et al., 2021; Medaglia, Harvey, et al., 2018). Specifically, if the region is responsible for a large amount of integration or segregation within the network, this could cause tension with its ability to effectively process language demands. Thus, higher BC in the region would correspond to worse performance on language tasks.

Moreover, we expected relationships between BC at the rPTr and language processing. Prior neuromodulation studies have focused on naming as the main outcome measure (Hamilton et al., 2010; Naeser et al., 2004; Naeser, Martin, Nicholas, Baker, Seekins, Kobayashi, et al., 2005), and we might expect the integrative or segregative capabilities to be important in this aspect of language. Suppressing the rPTr with inhibitory neuromodulation is associated with lower phonological naming errors (Harvey et al., 2019). Inhibiting the rPTr may reflect a shift of recruitment to the nearby right pars opercularis (rPOperc), which is thought to be associated with phonological processing (Turkeltaub et al., 2011). Thus, the capacity of the rPTr to integrate or segregate may correspond to phonological naming errors in PWA. Notably, Harvey et al. (2019) did not find a relationship between inhibitory neuromodulation of the right rPTr and semantic errors. Therefore, it is possible that the rPTr has a role in phonological processing, which could be related to the structural properties of the region. Harvey et al. (2019) suggests the rPTr specificity to phonological errors could be that inhibiting the region increases the relative recruitment of nearby regions that are more efficient at language processing, such as the rPOperc.

In the current study, we aimed to elucidate the role of the rPTr by investigating structural network differences in this region between PWA and neurotypical controls. In light of prior evidence that found consistent functional activity at the rPTr in PWA but not in controls (Turkeltaub et al., 2011), we predicted higher BC in PWA at rPTr. To investigate whether individual differences in BC relate to language deficits, we tested for a relationship between BC and overall aphasia severity, as well as performance on a naming test. The naming test measures included assessing naming accuracy, the proportion of phonological errors, and the proportion of semantic errors. Building off the work from Harvey et al. (2019), we predicted that the BC of the rPTr will correspond to phonological naming errors and not semantic naming errors. Finally, we also tested these hypotheses in two control sites: rPOperc and the right occipital lobe. We used the rPOperc as a control site to determine whether changes in BC are specific to the rPTr or extend to nearby regions as well. Given that functional activity was higher in the rPTr in PWA, we expect BC shifts to remain focal to the rPTr. Moreover, we also used the right occipital lobe as a non-language homotopic control site to determine if BC changes occur in the entire brain for PWA or only in language homologues. Finally, as an exploratory aim, we examined whether BC relates to global topological properties in PWA. We chose to investigate the use of modularity, a measure of community structure in a network (Rubinov & Sporns, 2010), as our measure of global topological properties due to prior research showing shifts in modularity after stroke and relationship with behavior (Siegel et al., 2016, 2018). Moreover, BC also reflects the capacity of a region to integrate and segregate communities in the brain. Therefore, we are interested in examining whether global changes in community organization correspond to local region-level changes in community interaction.

## MATERIALS AND METHODS

### Participants

We used data from the University of Pennsylvania (47 participants, 21 PWA) and Georgetown University (76 participants, 39 PWA) to investigate our hypotheses. In total, we had 60 PWA (age (Mean = 59.9, *SD* = 9.17; months post stroke average = 54.42, *SD* = 51.8; 22 female) and 62 neurotypical controls (age = 44.65, *SD* = 20.05; 31 female). All stroke participants were at least 6 months post-stroke and suffered only a single stroke. All controls from Georgetown University dataset completed the Montreal Cognitive Assessement (MoCA) and scored higher than 26. All participants were native English speakers and were right-handed. All procedures were approved in a convened review by the respective institution’s Institutional Review Board and were conducted in accordance with the guidelines of the Institutional Review Board/Human Subject Committee. All participants provided informed consent before data collection.

### Neuroimaging Procedure

**Figure 1.**
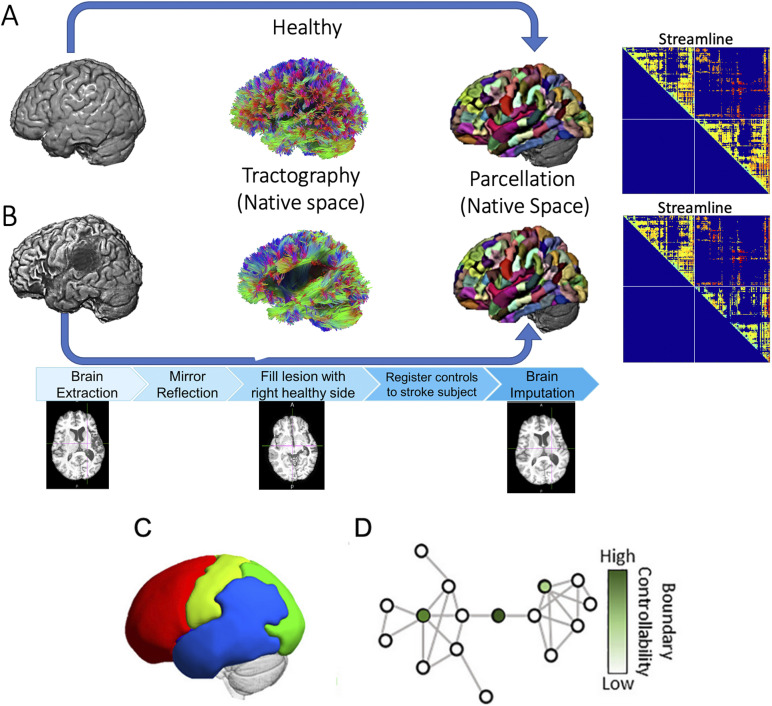
Overview of the study methods. (A) Processing scheme for healthy controls. DiJusion tractography was computed in subjects’ native space, and the Lausanne multiscale parcellation fit subjects’ anatomic T1 images. Connectomes were defined based on streamline counts of the edges connecting each region pair and advanced to analyses. (B) The processing scheme for persons with aphasia was the same as that of the healthy controls, with an additional preprocessing step. Specifically, the anatomic T1 image was imputed using the stroke subject’s right hemisphere and healthy subjects’ data to estimate the prelesion T1 anatomic image. The parcellation was computed on this imputed anatomic image to guide connectome extraction through the same regions as the controls. (C) Community detection within each participant. We applied a community detection algorithm multiple times within each subject, then obtained a consensus partition. The figure shows an example of a final community partition for a single subject, where each color represents the regions assigned to a specific community. (D) Boundary controllability were computed for each node (brain region) in the network for each individual. Each node received a rank representing its strength of control within the individual. Figure adapted from Medaglia, Harvey, et al. (2018).

**Figure 2.**
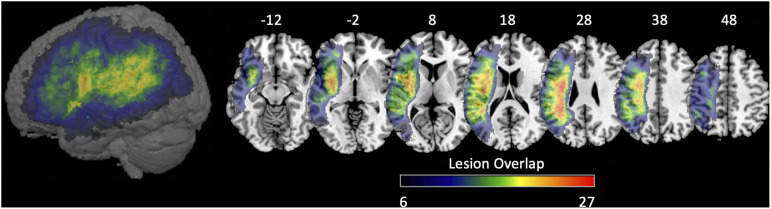
Voxelwise lesion overlap map of 60 persons with aphasia. Color bar references the number of patients with a lesion at a given voxel. Slices are MNI coordinates.

We acquired 3.0T T2 and diffusion-weighted images (DWI) or diffusion spectrum imaging (DSI) for all subjects along with a T1-weighted 1 mm resolution MPRAGE anatomical scan at each scanning session as part of a larger imaging protocol. We used a popular preprocessing pipeline to clean and process the diffusion data (Tournier et al., 2019), which includes standard noise-correction procedures for motion, eddy correction, and inhomogeneity. To select the anatomical ROIs, we used a modified version of the Lausanne multiscale atlas (Cammoun et al., 2012) with 234 brain regions estimated in each subject’s native space. We used this parcellation for consistency with studies demonstrating stability of global network properties at this resolution (Bonilha et al., 2015; Meskaldji et al., 2013; Termenon et al., 2016). Lesions cause sharp changes in signal intensity at their boundaries. These changes can obscure smaller differences associated with the gray/white matter border, which is necessary to fit an accurate cortical parcellation. Thus, a brain imputation step was done to ensure that the parcellation can properly distinguish between white and gray matter boundaries, which is necessary for the parcellation to fit onto the individual’s brain. All imputation steps are done with only the T1w image and do not impact the streamline connectivity, and we did additional analysis to test the effects on the extracted connectomes. Additionally, the Lausanne parcellation will be distorted if cortical areas are missing. Briefly, the imputation procedure mirrored the contralesional hemisphere into the lesion of the ipsilesional hemisphere. This allowed us to fill in the damaged tissue with highly similar morphologic features to the damaged tissue. Through this process, we used similar tissue to help differentiate gray and white matter boundaries to facilitate segmentation and fit the parcellation with minimal bias. The tissue boundaries in the imputed brain were enhanced using a joint image fusion procedure weighted with values from an age-matched sample of healthy controls. After imputation, we manually checked each parcellation with the imputed brain volume to ensure all parcels are correctly registered to the brain volume. See Figure 1 for a methodological overview and Figure 2 for lesion overlap of patients. Full scanning parameters, diffusion tractography methods, and detailed imputation methods are included in the Supplementary Material, available at https://doi.org/10.1162/nol.a.11.

We then constructed a structural brain network (adjacency matrix) using the Lausanne brain regions as the basis for nodes and the presence of streamlines between regions as the basis for edges. Once the adjacency matrix was constructed, we computed the BC for each brain region using the same approach as Medaglia, Huang, et al. (2018) and Medaglia et al. (2021). To summarize, we used MATLAB to compute BC in each brain region by partitioning the brain into modules by maximizing the modularity quality function (Newman, 2006) using a Louvain-like (Blondel et al., 2008) locally greedy algorithm (Jeub et al., 2011). To account for near degeneracies in determining the modularity landscape, we optimized the algorithm 100 times (Good et al., 2010). Similar to Medaglia et al. (2021), we used a fully weighted streamline network and partition within each individual. To find a consistent partition threshold, we obtained partitions within each of the 100 optimizations per subject at each value of *γ* from 1.0 to 4.0 in increments of 0.1. We then calculated a mean z-Rand coefficient for each subject across values of *γ* and identified the peak z-Rand. We then used that *γ* value for each subject for the remainder of the analysis in this study. High-ranking boundary controllers were identified as the highest-ranking set of boundary regions between modules, and the remaining boundary regions were found within modules in the network. We utilized ranked BC because computing BC incorporates the computation of eigenvectors, the exact scalar values of which can be unreliable due to computational precision when applied to brain networks and prohibit comparisons between subjects (Gu et al., 2015). For more details on the computation of BC, please see Medaglia et al. (2021), Medaglia, Huang, et al. (2018), and Gu et al. (2017).

To measure modularity, we found the optimal subdivision of the network such that within-group edges were maximized and out-group edges were minimized (Newman, 2006). To do so, we used the modularity function from the Brain Connectivity Toolbox (Rubinov & Sporns, 2010). Using each participant’s structural adjacency matrix and *γ* from the BC calculation described above. We then computed Newman’s *Q* over 100 iterations and used the average as our value of modularity.

### Language Tasks

All PWA completed the Western Aphasia Battery (WAB; Kertesz, 1982), and we used the Aphasia Quotient (WAB-AQ) score for our measure of general language deficits. The WAB-AQ score includes performance on subtests of spontaneous speech, auditory verbal comprehension, repetition, naming, and word finding. A subset of participants (*n* = 50) also completed a shortened version of the Philadelphia Naming Test (PNT; Lacey et al., 2017). This test asks participants to name black and white drawings. We used overall accuracy and proportion of phonological and semantic errors to investigate different aspects of language processing. We coded errors in line with prior research that has used the PNT (Roach et al., 1996).

### Data Analysis

We had two sets of planned analyses to test the hypotheses that BC differed in PWA relative to controls, and that BC was related to language deficits in PWA. We also had an exploratory analysis to examine whether BC was related to modularity. To investigate if BC at the rPTr differed between PWA and controls, we compared the BC rank across participants in each group using an ordinal logistic regression test to examine if there was an interaction between group (PWA and controls) and region (rPTr, rPOperc, right occipital; see Supplementary Figure S1 for distribution of BC across sites and group). We hypothesized a significant interaction between group and region, and used Wilcoxon non-parametric *t* tests to test our prediction that only the rPTr would show a significant difference in BC between groups. An assumption of parametric tests is that all values are independent of one another, which is violated by using ranked values of BC across regions within the same individual. Thus, we chose to use a non-parametric test for our analysis.

To test if BC at the rPTr relates to language processing in PWA, we ran a series of linear regression models to compare the relationship between BC and language task performance (WAB and the PNT). First, we z-scored each measure and tested if each measure (i.e., WAB, PNT overall accuracy, PNT semantic errors, PNT phonological errors) related to BC within the PWA. For this series of analyses, we only expected phonological errors to relate to BC.

For the exploratory analysis, we ran a correlation between BC and modularity and then included modularity as a covariate in our regression models. We performed all statistics using the R programming language (R Core Team, 2020). We used a critical *p* value of 0.05 to determine statistical significance for all statistical analyses. We applied a false discovery rate (FDR) to the *p* values for the Wilcoxon non-parametric *t* tests that were done to understand interaction effects further because these tests were dependent on the omnibus test (García-Pérez, 2023). *P* values for the Wilcoxon *t* tests are the FDR-corrected values.

## RESULTS

**Figure 3.**
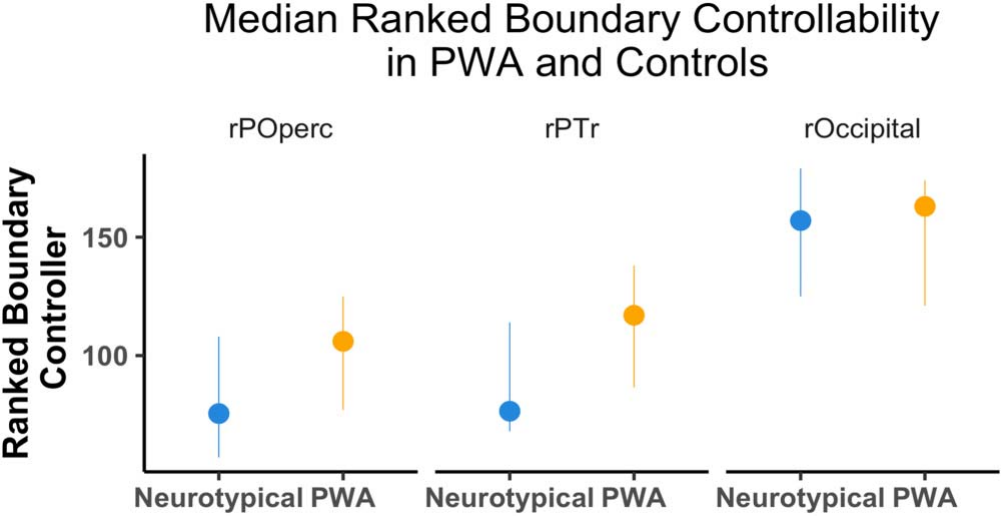
Boundary controllability (BC) at right pars opercularis (rPOperc), right pars triangularis (rPTr), and right occipital (rOccipital) in persons with (PWA) and neurotypical participants. PWA had a statistically significantly higher median BC at the rPTr compared to controls. However, PWA and controls had no significant median BC diJerence at the rPOperc or rOccipital. Bars represent 95% confidence interval.

We performed an ordinal logistic regression test to compare the BC contrasts between PWA and controls at each of our regions. The interaction between group and region was statistically significant. The difference in BC at the rPTr between PWA and controls was larger than the BC difference seen in the right lateral occipital (*t*(4) = −2.02, *p* = 0.04) and in the rPOperc (*t*(4) = −0.45, *p* = 0.65). We then ran a Wilcoxon non-parametric *t* test to compare the BC in PWA and controls at rPTr. Note, these tests are FDR-corrected *p* values. The results were statistically significant (*W* = 1,403.5, *p* < 0.05; see Figure 3) with PWA (median = 117.0, median absolute deviation [*MAD*] = 71.16) having a higher BC than controls (median = 76.5, *MAD* = 61.53). As a post hoc test, we also asked if BC was different between groups in our control sites. For the right pars opercularis, we found no statistical difference (*W* = 1,157, *p* = 0.12) between PWA (median = 105.5, *MAD* = 71.9) and controls (median = 76.5, *MAD* = 73.39). We also did not find a statistical difference (*W* = 1,992.5, *p* = 0.5) in the right lateral occipital between PWA (median = 163, *MAD* = 58.56) and controls (median = 157, *MAD* = 63.01). In summary, we found that the rPTr had a higher BC in PWA relative to controls and that the magnitude of this difference was larger than the magnitude observed in the rPOperc and the right occipital regions.

As an additional post hoc analysis, we also examined the relationship between BC and total lesion volume (TLV) and the percent damage to the left pars triangularis. We did not find a statistically significant correlation between TLV and BC (*r*(58) = 0.14, *p* = 0.32) or with damage to the left pars triangularis (*r*(58) = −0.01, *p* = 0.91). Thus, lesion size and damage to the homotope did not relate to the BC changes we see in PWA. Finally, we also examined the relationship between BC and node strength (Rubinov & Sporns, 2010), which is the sum of all edge weights emanating from the node. We used this measure to determine if our findings were due to weighted connections to the rPTr, or due uniquely to the more complex intermodular role measured by BC. Critically this relationship was not significant in PWA (*r*(48) = 0.15, *p* = 0.32) or in the controls (*r*(60) = −0.08, *p* = 0.56). Thus, differences in BC is not due to quantity of connections, but rather how those connections allow the region to integrate/segregate brain regions.

One concern with our approach is that the imputation procedure may have impacted the quality of results. To test this, we paired 31 controls with an age-matched lesion from the PWA, and then registered the lesion to their diffusion image. We then fit each brain into the parcellation, including the imputation step to account for the lesion. We then re-ran the imaging procedure for each “lesioned” control through our pipeline, including the imputation step. Finally, we recalculated the BC of the rPTr in the controls with the lesion-imputed network. The Wilcoxon non-parametric *t* test was not statistically significant (*W* = 515.5, *p* = 0.63; see Supplementary Figures S2 and S3 for results). We noted in this analysis that the median value of the distribution decreased despite a null change in the overall distribution. Upon visual inspection, the BC ranked values exhibit some shift in central tendency. However, the data may still be drawn from the same underlying distribution suggested by the null Wilcoxon test. Thus, while some uncertainty in the exact node ranks may be contributed by the effects of the imputation procedure on the final parcellation, these effects are unlikely to drive the primary results. Any bias in the median toward lower values due to the imputation effects on the parcellation would run contrary to our finding that BC increases in PWA.

**Figure 4.**
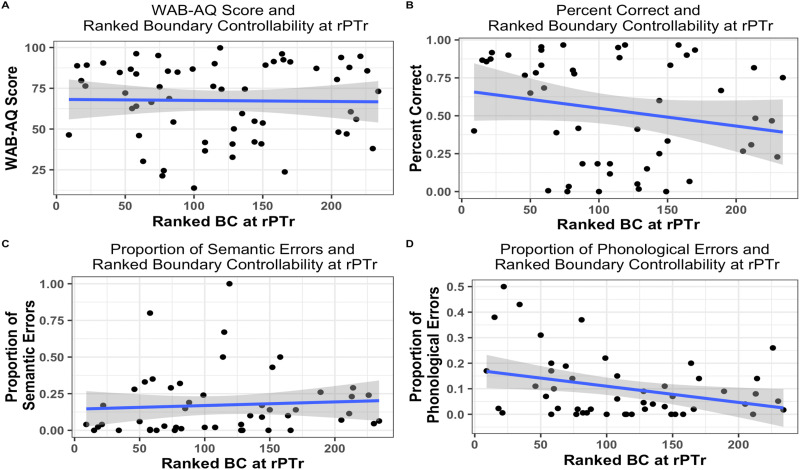
Scatterplots depicting the relationship between BC and behavioral measures.

To test the relationship between BC and language performance, we ran four linear regression models with task performance (WAB-AQ, PNT overall accuracy, and error proportion) as the dependent variable and BC at the rPTr as the independent variable (see Table 1 for full details). For the WAB-AQ model, the relationship between WAB (mean = 67.4, *SD* = 23.33) and BC (*R*^2^ = 0.0003, *F*(1, 57) = 0.02, *p* = 0.9) was not significant. For the PNT models, there was no significant relationship between BC and overall naming accuracy (mean overall naming accuracy = 53%, *SD* = 34%, *R*^2^ = 0.04, *F*(1, 48) = 2.25, *p* = 0.14) or proportion of semantic errors (mean proportion of semantic errors = 17%, *SD* = 22%, *R*^2^ = 0.005, *F*(1, 48) = 0.24, *p* = 0.62). The relationship between BC and proportion of phonological errors was significant and negative (mean proportion of phonological errors = 10%, *SD* = 12%, *R*^2^ = 0.1, *F*(1, 48) = 5.59, *p* = 0.02, BC *b* = −0.0006, *t*(48) = −2.36, *p* < 0.05; see Figure 4). To determine if the results were due to lesion volume, we ran a follow-up model with TLV as a covariate. Even with TLV in the model, BC still accounted for a significant amount of the variance in phonological errors (*b* = −0.0006, *t*(48) = −2.47, *p* = 0.02). We also ran a model with BC at the rPOperc to determine if the control site also related to phonological errors in overt naming. The overall model was not statistically significant *R*^2^ = 0.06, *F*(1, 48) = 3.26, *p* = 0.07, BC *b* = −0.0005, *t*(48) = −1.87, *p* = 0.07). These results did not change when we included TLV in the model (*b* = −0.0004, *t*(48) = −1.72, *p* = 0.09). Due to the trending *p* value in the rPOperc model, we ran a post hoc test with the BC of the rPTr and rPOperc to determine if the unique variance of the rPTr is significantly related to phonological naming errors when controlling for the BC of the rPOperc. The BC of the rPTr was not significantly related to phonological naming errors when the BC of the rPOperc was included in the model (*b* = −0.0005, *t*(48) = −1.63, *p* = 0.1).

**Table 1.** Right pars triangularis regression model results

Measure	*R* ^2^	*F* statistic (*df*)	Beta estimate
WAB-AQ	0.0003	0.02 (1, 57)	−0.006
Overall accuracy	0.04	2.25 (1, 48)	−0.001
Proportion of semantic errors	0.005	0.24 (1, 48)	0.0002
Proportion of phonological errors	0.1	5.89 (1, 48)*	−0.0006*
Proportion of phonological errors (TLV covariate)^a^	0.11	6.21 (1, 48)*	−0.0006*

^a^ Total lesion volume (TLV) was included as a covariate in the model.

WAB-AQ = Western Aphasia Battery—Apahsia Quotient (Kertesz, 1982).

* *p* < 0.05.

For our exploratory analysis, we examined how global topological properties interacted with the local properties of the rPTr. There was no correlation between modularity and BC at the rPTr (*r*(58) = 0.08, *p* = 0.53). When we included modularity as a covariate in our regression model between BC and phonological naming errors, we found no relationship between modularity and errors (*b* = 0.009, *t*(48) = 0.33, *p* = 0.73) and the relationship between BC and naming errors was still significant (*b* = −0.0006, *t*(48) = −2.36, *p* = 0.02).

## DISCUSSION

The broad aim of our study was to understand the structural role of the rPTr in PWA and how that role might relate to language processing. We tested the hypothesis that left hemisphere stroke would result in structural shifts in the network role of the rPTr in PWA relative to healthy controls. We predicted the topological properties of the rPTr would relate to phonological processing. Overall, the data supported these hypotheses.

Specifically, PWA had a higher ranked BC at the rPTr relative to controls, and the number of phonological naming errors the patients made was related to the rank of BC in the rPTr. This relationship was significant even when controlling for lesion volume and modularity.

Moreover, we did not find a relationship between BC and aphasia severity, overall naming accuracy, and semantic naming errors. Finally, we did not find evidence that these structural changes or topology-behavior relationships exist in the rPOperc or the lateral occipital control sites. Behaviorally, we found that the rPTr had a negative relationship with the proportion of phonological naming errors and overall naming accuracy but no relationship with overall aphasia severity or semantic naming errors.

Differences in BC between PWA and controls suggest that a structural disruption can cause shifts in network properties in regions in the contralesional hemisphere. Prior work demonstrates that topological changes occur after stroke, such as the number of communities in the brain depending on the nature of the lesion (Gratton et al., 2012; Griffis et al., 2019, 2020; Siegel et al., 2016, 2017, 2018). As a result, right hemisphere regions have the potential to take on additional network responsibilities at the structural level (Medaglia et al., 2022). Our findings suggest that at least part of this shift is to the rPTr in PWA based on changes in structural network organization. Further evidence for a change in this region comes from prior work that the rPTr has higher functional activity in PWA relative to controls (Turkeltaub et al., 2011). We propose that this increased activity could be due to the new theoretical dynamic role that changes the structural connectivity of the region occupied in the network. Specifically, when the rPTr changes to serve a potentially more prominent role in integrating or segregating activity between regions. Moreover, our results suggest that this shift is not due to damage to the homotopic region (i.e., left pars triangularis) or the size of the lesion. Speculatively, BC changes could be due to the loss of specific edges in the left hemisphere. This could heighten the importance of edges connecting to the rPTr, which leads to an increase in the BC of the region. Future work is needed to determine how the loss of specific edges may cause the network role of the rPTr to shift. Notably, the relationship between phonological errors and BC was also negative in the rPOperc, which could suggest that the importance of integration or segregation is not specific to the rPTr. More work will need to examine how the rPOperc could also be important for language network reorganization.

In addition to prior research in healthy populations, the current study demonstrates that BC at the rPTr is an important aspect of language recovery in PWA. Prior research suggests that individual differences in the capability of the left pars triangularis to integrate or segregate brain regions is related to cognitive selection demands (Medaglia et al., 2021; Medaglia, Huang, et al., 2018). Specifically, when the left pars triangularis was higher in BC, neurotypical participants were slower to respond on a selection task (Medaglia, Huang, et al., 2018). Based on this work and our results, we hypothesize that potential integration (or strategic segregation) between two or more communities may provide more information when processing language demands. For PWA, this integration or segregation leads to fewer phonological naming errors and better naming performance. We speculate that the integrative role of BC could be especially important for PWA, where the capacity of the rPTr to integrate across the entire brain is important for language demands to be met.

Future work could use functional connectivity to determine how co-activity between the rPTr and the residual language network corresponds to the relationship between BC at rPTr and language recovery. This kind of study would also help establish if the rPTr is a stronger integrator or segregator, which we cannot dissociate with structural connectomes.

Our findings are also consistent with prior research that revealed that right hemisphere activity during language processing can lead to relatively better performance in PWA. While prior studies have found that functional and structural changes in the right hemisphere can relate to better language performance in PWA (Leff et al., 2002; Skipper-Kallal et al., 2017a, 2017b; Xing et al., 2016), even specific to phonological processing (Blank et al., 2003; Harvey et al., 2019; Xing et al., 2016), no studies have found evidence of this in the rPTr. Rather, most research associates functional activity in this region with worse language performance (Cao et al., 1999; Postman-Caucheteux et al., 2010) or finds no relationship (Skipper-Kallal et al., 2017a, 2017b). The mixed results related to the functional activity of the rPTr and language performance could be related to BC. Specifically, prior studies have demonstrated a robust link between structure and function (Alexander-Bloch et al., 2013; Hagmann et al., 2008; Honey et al., 2009; Suárez et al., 2020), which we suggest could extend to the relationship between structural properties (e.g., BC) and functional activity.

Based on this work and our current results, we hypothesize that potential functional integration (or segregation) between two or more communities may provide more information when processing language demands. For PWA, this integration or segregation may lead to fewer phonological naming errors and better naming performance. The integrative role of BC could be especially important for PWA, where the capacity for left hemisphere regions to interact with right hemisphere regions may improve language processing. Conversely, higher BC at the rPTr could improve integration among right hemisphere regions critical for language processing in PWA, such as the rPOperc and the right temporal lobe. Future work could use functional connectivity to determine how co-activity between the rPTr and the residual language network corresponds to the relationship between BC at rPTr and language recovery. This would also help establish if the rPTr is a stronger integrator or segregator, which we cannot dissociate with structural connectomes.

From a clinical perspective, BC could also mediate which patients respond to neuromodulation of the rPTr. For example, prior studies have noted that only some patients improve in naming performance after inhibitory neuromodulation to the rPTr (Hamilton et al., 2011; Martin et al., 2009; Medaglia et al., 2021; Medaglia, Harvey, et al., 2018; Turkeltaub, 2015; Turkeltaub et al., 2012). Individual variation in BC at the rPTr could potentially explain some of these mixed findings. For instance, if BC is weaker at rPTr, inhibiting the region could prevent it from inefficiently engaging in language processing, which allows other right hemisphere regions to assist in meeting language task demands. Conversely, inhibiting the rPTr when it is higher in BC could prevent the residual language network and right hemisphere regions from communicating. More work is needed to determine if BC can help serve as a predictor of neuromodulation efficacy in PWA.

Our study also aligns with past findings that BC is an important predictor of language processing in healthy controls. Prior research suggests that individual differences in the capability of the left pars triangularis to integrate or segregate brain regions is related to cognitive selection demands (Medaglia et al., 2021; Medaglia, Huang, et al., 2018). Specifically, when the left pars triangularis was higher in BC, neurotypical participants were slower to respond on a selection task (Medaglia, Huang, et al., 2018). In PWA, researchers have also proposed that the rPTr is not involved specifically in language processing in PWA but instead mediates task difficulty (Brownsett et al., 2014). Our findings in the current study that BC at the rPTr was related to phonological naming performance is contrary to this interpretation of the rPTr in PWA. However, future work could examine whether task demands in language relate to BC in PWA, similar to what has been found in the homotopic region in healthy participants (Medaglia et al., 2021; Medaglia, Huang, et al., 2018).

Our exploratory aim of examining the relationship between BC and modularity also demonstrated that BC changes in the rPTr occur at the local region level and are not due to global shifts in modular topology. We focused on modularity because of prior evidence that disrupted modularity corresponds to language deficits in post-stroke patients (Siegel et al., 2016, 2018). Critically, BC quantifies the extent to which a node mediates between major modules in the brain. Thus, it was possible that higher BC at the rPTr might relate to this aspect of overall modular organization. Since we did not find that relationship, it is possible that the ability of the region to integrate or segregate functional activity can be independent from global community organization. Thus, there are possibly unique properties of the rPTr that allow it to take on a larger role in community integration after stroke. One possible reason for the change in BC is the loss of structural connections from the rPTr to other brain regions that selectively increase its role in the control hierarchy among modules. For example, specific pathways might be important for a region to be higher in BC relative to another region. When some of these pathways and regions are lost as a result of left hemisphere stroke, the existing pathways and regions become even more important relative to the rest of the remaining network because local losses of regions and connections also change the overall network topology (Betzel & Bassett, 2017; Medaglia et al., 2022; Moon et al., 2015). The increased BC in the rPTr could be a symptom of the total damage to the left hemisphere. This increase in BC is therefore less likely a specific effect of rPTr gaining importance and more likely due to the loss of other regions that result in the rPTr gaining more relative centrality among remaining modules in the network whose distribution was affected by the lesions. The specific mechanisms that drive this result are beyond the scope of our current study. We speculate this could be due to a deeper relationship between local (i.e., edge-level) and global (e.g., modularity) properties of the rPTr, and recommend that large-scale cross-sectional and simulation studies could begin to answer this question.

Overall, our study provides a new technique for exploring anatomical changes after a stroke. NCT gives unique insights into the theoretical dynamic role of a region and how it may shift after damage to the network. Furthermore, this work suggests the role of the rPTr in stroke by demonstrating that individual differences that exist in structural role involvement, but are not explained by overall differences in network topology, correspond to the dysfunction associated with the region during language processing. The capacity of the rPTr to fulfill the role of an integrator or segregator in an individual’s network also relates to their phonological naming abilities. As with similar work on the right hemisphere in aphasia recovery, phonological processing seems to be localized to right hemisphere regions. We propose that this individual variation in BC could be a useful marker for aphasia outcomes and treatment, and its role in neuroplasticity should be further examined.

## ACKNOWLEDGMENTS

The authors would like to acknowledge Elizabeth Lacey for data collection and Mackenzie Fama and Ayan Mandal for data collection and scoring.

## FUNDING INFORMATION

Peter E. Turkeltaub, National Institutes of Health (https://dx.doi.org/10.13039/100000002), Award ID: R01DC014960. Peter E. Turkeltaub, National Institutes of Health (https://dx.doi.org/10.13039/100000002), Award ID: KL2TR000102. Peter E. Turkeltaub, Doris Duke Charitable Foundation (https://dx.doi.org/10.13039/100000862), Award ID: 2012062. John D. Medaglia, National Institutes of Health (https://dx.doi.org/10.13039/100000002), Award ID: 1-DP5-OD-021352–01. Roy H. Hamilton, National Institute on Deafness and Other Communication Disorders (https://dx.doi.org/10.13039/100000055), Award ID: 5-R01-DC-016800.

## AUTHOR CONTRIBUTIONS

**Harrison Stoll**: Conceptualization: Lead; Data curation: Equal; Formal analysis: Lead; Investigation: Lead; Writing – original draft: Lead; Writing – review & editing: Lead. **Apoorva Kelkar**: Data curation: Equal; Methodology: Equal. **Peter E. Turkeltaub**: Funding acquisition: Equal; Supervision: Supporting; Writing – review & editing: Supporting. **Roy H. Hamilton**: Funding acquisition: Equal; Supervision: Supporting; Writing – review & editing: Supporting. **Branch Coslett**: Writing – review & editing: Supporting. **John D. Medaglia**: Conceptualization: Supporting; Formal analysis: Supporting; Methodology: Equal; Supervision: Lead; Visualization: Supporting; Writing – original draft: Supporting; Writing – review & editing: Equal.

## DATA AVAILABILITY STATEMENT

All preprocessed connectomes and behavioral data are available on GitHub at https://github.com/CogNeW/Stoll_et_all2025.

## Supplementary Material



## TECHNICAL TERMS

Network control theory (NCT): A framework analyzing how to steer complex networks toward desired states using external inputs.
Boundary controllability (BC): The ability to control a system’s state by influencing only its boundaries or external nodes.
Modularity: Measures how well a network divides into communities by comparing actual connections to random ones; often denoted as Q.
Community detection: Finding groups of nodes in a network that are more connected to one another than to the rest.
Z-Rand coefficient: A metric to compare how similar two groupings of nodes are, accounting for random overlaps.
