# Supplementary material for: Lesion-Induced Changes to the Network Controllability of the Right Pars Triangularis in Aphasia
Source: Neurobiol Lang (Camb). 2025 Sep 2;6:nol.a.11. doi: 10.1162/nol.a.11 (PMC12404640; doi:10.1162/nol.a.11)
Supplement: Supplementary file 1 [file nol-6-1-11-s001.pdf]

Supplementary Materials

May 29, 2025

1 Image Acquisition

1.0.1 Georgetown University Neuroimaging Pipeline

Diffusion-weighted images (DWI) were acquired on a Siemens 3.0T Magnetom Trio for all 76 participants along with a T1-weighted 1mm resolution MPRAGE anatomical scan at each scanning session as part of a larger imaging protocol. We used a high-angular resolution diffusion imaging(HARDI) acquisition scheme with a maximum b-value of 1,100 (80 dirs, 10 b = 0; 10 b = 300; 60 b = 1100) and a 2.5 mm isotropic voxel size. We used an ACPC aligned acquisition of 55 axial slices with the following parameters: repetition time (TR) = 7.5 s; echo time (TE) = 87 ms; field of view (FoV) = 240 x 240 mm, matrix = 96, total acquisition time of 10:00. MPRAGE scans were collected with TR = 1900 ms, TE = 2.52 ms, 176 sagittal slices with 0.9mm slice thickness, FoV = 240 x 240, matrix = 256, inversion time (TI) = 900ms and flip angle = 9°, total acquisition time of 5:34.

1.0.2 University of Pennsylvania Neuroimaging Pipeline

DWI were acquired on a 3T Prisma scanner for all XXX subjects along with a T1-weighted 1mm resolution MPRAGE anatomical scan. We used two different protocols for the 47 participants. Eleven patients received HARDI acquisition scheme with a maximum b-value of 3000 (128 dir) and a 2 mm isotropic voxel size. We used an ACPC aligned acquisition of 74 transversal slices with the following parameters: TR = 5.2 s; TE = 82 ms; FoV = 232 x 232 mm, total acquisition time of 11:26 seconds. MPRAGE scans were collected with TR = 1380 ms, TE = 3.5 ms, 175 sagittal slices with 1 mm slice thickness, FoV = 256 x 256, TI = 1100 ms and flip angle = 7°, total acquisition time of 5:51. The remainder of the patients and all of the neurotypical participants received a different protocol. Specifically, they received a HARDI acquisition scheme with a maximum b-value 5000 (257 dir) and an isotropic voxel size of 2.4 mm. An ACPC aligned acquisition of 52 axial slices with the following parameters: TR = 5 s; TE = 138 ms; FoV = 232 x 232 mm. MPRAGE scans were collected with a TR = 2400 ms, TE = 2.2 ms, 208 sagittal slices with a .88 mm slice thickness, FoV = 231 x 231, TI = 1000 ms, and flip angle = 8°, total acquisition time of 19:30.

1.1 Brain Imputation and Parcellation

The brain imputation step was done to ensure that the parcellation can properly distinguish between white and grey matter boundaries, which is necessary for the parcellation to fit onto the individual's brain. All imputation steps are done with only the T1w image and do not impact the structural connectivity measures used for the connectome. We first imputed a version of each stroke subject's premorbid brain. Lesions were initially traced on each subject's anatomical T1 image by an experienced cognitive neurologist (coauthor PET, HBC). The contralateral hemisphere was then flipped and registered to the lesioned hemisphere, and the lesioned voxels were filled in. We then corrected for sharp changes in signal intensity. We used ANTs' joint image fusion procedure, where images more similar to a combination of the values from all the healthy images around the voxel of interest receive more weight (similar to multi-atlas label fusion). We searched for the optimal number of healthy brains and radius of similarity around each voxel, and found optimal outcomes with 22 healthy brains and a radius of 1 (i.e., a single layer of voxels around each voxel is used to check the similarity between images and assign weights to healthy images). We inspected the resulting imputed images for artifacts and found none. The gyri and sulci of each subjects' imputed image followed the gyri and sulci of their flipped RH image without visible deviation. We performed all imputation procedures in ANTs (v. 2.2.0) (imputation). Before any processing, all images were skull-stripped (antsBrainExtraction.sh), corrected for magnetic field inhomogeneity (N4BiasFieldCorrection), and denoised with an edge preserving algorithm (PeronaMalik, denoising amount: 0.7, iterations: 10). We added the lesion mask back to the brain mask after skull-stripping to ensure that the lesion area was included in the imputation. We conducted all registrations using the SyN non-linear algorithm (Avants et al., 2008) with cost function masking to remove the lesion mask from consideration during the registration computations (Brett et al., 2001).

1.2 Diffusion Tractography

We used MRtrix3 (Tournier et al., 2019) to denoise the diffusion images (function: dwidenoise -extent 9,9,9), correct for motion and eddy currents (function: dwipreproc), and correct for field inhomogeneity (function: dwibiascorrect). We then computed response functions for multiple tissues using the tissue information available in the DWI data (function: dwi2response dhollander). Finally, we computed fiber orientation distributions (FOD) via a multi-shell multi-tissue constrained spherical deconvolution (function: dwi2fod) (Jeurissen et al., 2014). To find the GM/WM tissue, we applied tissue classification to the imputed anatomical image (function: 5ttgen fsl) and brought the tissue information into DWI space after registering the original (lesioned and imputed) T1w image of the subject onto the mean b=0 image (function: antsRegistration, order: translation, rigid, SyN) and applying the transformations to the tissue types. We performed probabilistic anatomically constrained white matter tractography by seeding 15 million streamlines from the white matter based on estimated fiber densities (tckgen algorithm: iFOD2, step: 1mm, minlength: 10mm, maxlength: 300mm, angle: 45 degrees, seeding: dynamic, backtracking allowed, streamlines cropped at GM/WM border) (Smith et al., 2012). Spherical deconvolution informed filtering of tractograms (SIFT2) was conducted to determine the relative apparent fiber density

associated with each streamline Tournier et al. (2019); Smith et al. (2015a,b).

1.3 Parcellation

The edges of the structural connectome were generated by assigning streamlines to parcels of the Lausanne atlas at scales 125 scale and then multiplying each streamline by its respective cross-sectional multiplier derived by tcksift2. Inter-subject connection density normalization were achieved through scalar multiplication of each connectome by the subject's "proportionality coefficient" derived by SIFT2, denoted by μ, which represented the estimated fiber volume per unit length contributed by each streamline (Smith et al., 2015b, 2020). Overall, each edge value was directly proportional to the cross-sectional area of white matter connecting the two parcels.

2 Supplementary Figures

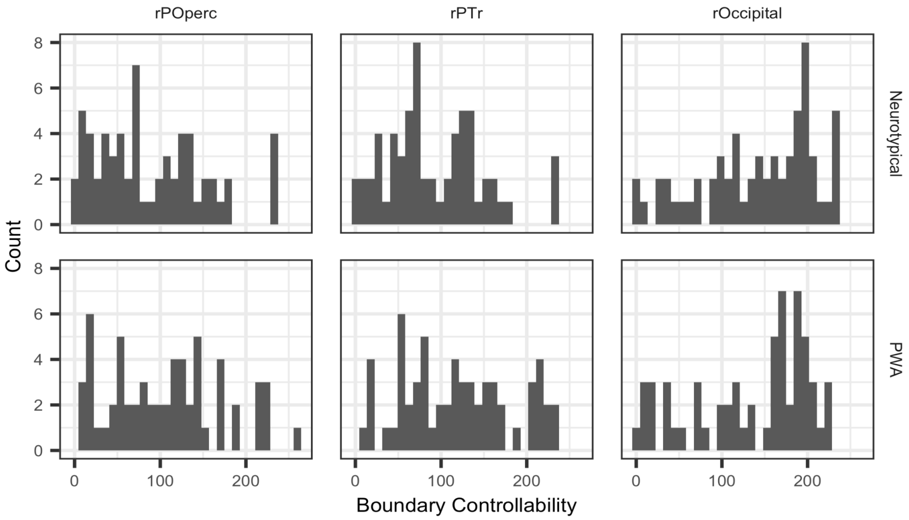

Figure 1: Histogram of boundary controllability for each site across across group

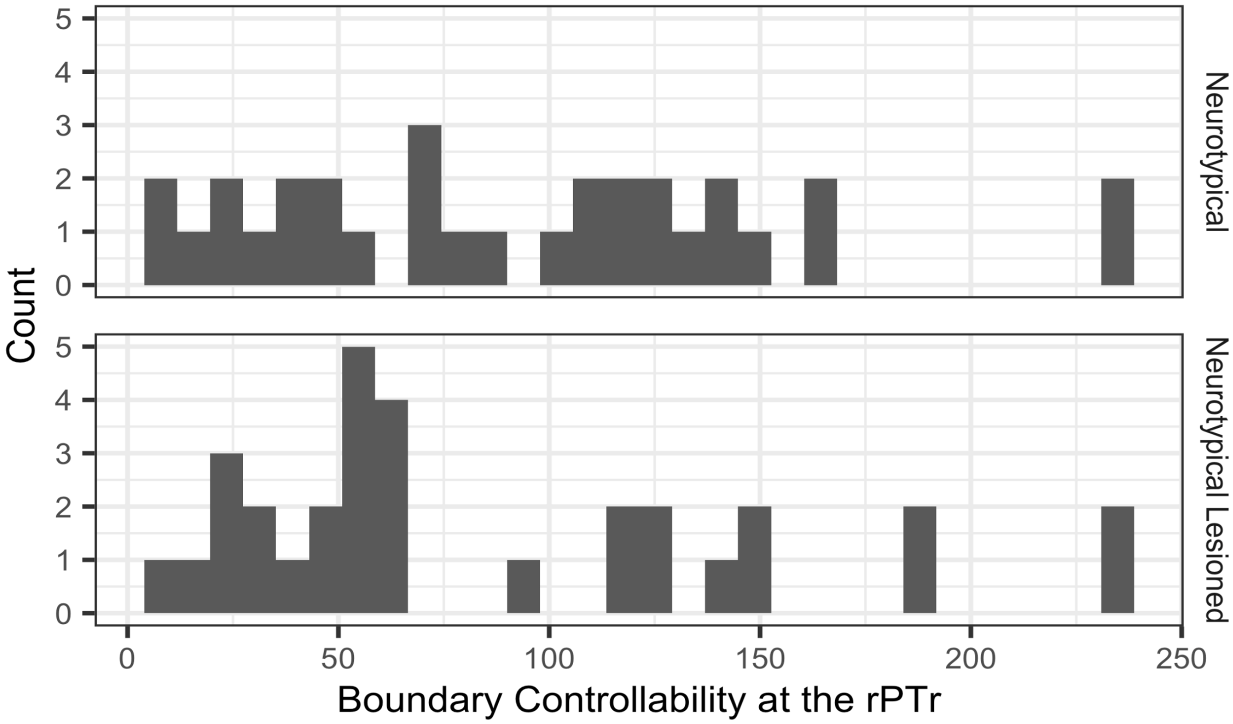

Figure 2: Histogram of the neurotypical and the virtually lesioned neurotypical controls boundary controllability at the rPTr.

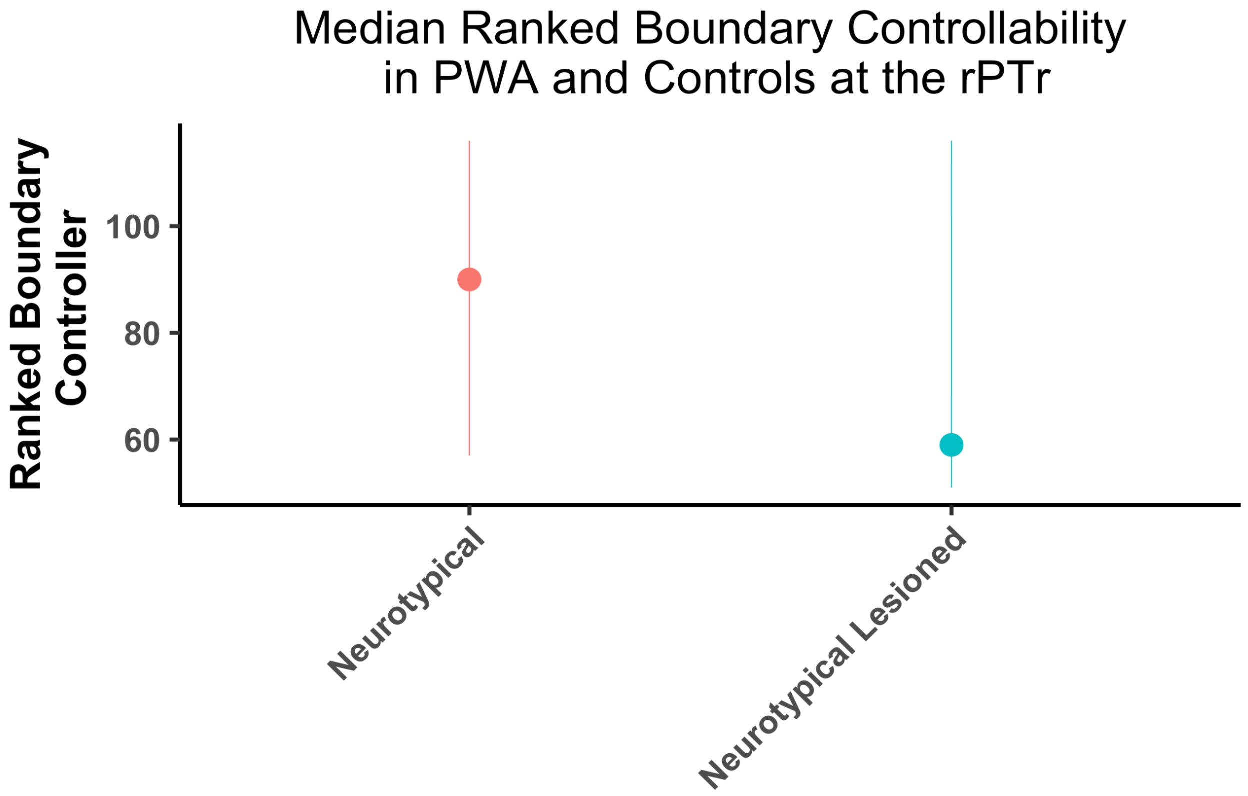

Figure 3: Boundary controllability at rPTr neurotypical and virtually lesioned neurotypical controls. Bars represent 95% confidence interval.

References

Avants, B. B., Epstein, C. L., Grossman, M., and Gee, J. C. (2008). Symmetric diffeomorphic image registration with cross-correlation: evaluating automated labeling of elderly and neurodegenerative brain. *Medical image analysis*, 12(1):26–41.

Brett, M., Leff, A. P., Rorden, C., and Ashburner, J. (2001). Spatial normalization of brain images with focal lesions using cost function masking. *Neuroimage*, 14(2):486–500.

Jeurissen, B., Tournier, J.-D., Dhollander, T., Connelly, A., and Sijbers, J. (2014). Multi-tissue constrained spherical deconvolution for improved analysis of multi-shell diffusion MRI data. *NeuroImage*, 103:411–426.

Smith, R., Raffelt, D., Tournier, J.-D., and Connelly, A. (2020). Quantitative streamlines tractography: Methods and inter-subject normalisation. Preprint, Open Science Framework.

Smith, R. E., Tournier, J.-D., Calamante, F., and Connelly, A. (2012). Anatomically-constrained tractography: improved diffusion mri streamlines tractography through effective use of anatomical information. *Neuroimage*, 62(3):1924–1938.

Smith, R. E., Tournier, J.-D., Calamante, F., and Connelly, A. (2015a). The effects of SIFT on the reproducibility and biological accuracy of the structural connectome. *NeuroImage*, 104:253–265.

Smith, R. E., Tournier, J.-D., Calamante, F., and Connelly, A. (2015b). SIFT2: Enabling dense quantitative assessment of brain white matter connectivity using streamlines tractography. *NeuroImage*, 119:338–351.

Tournier, J.-D., Smith, R., Raffelt, D., Tabbara, R., Dhollander, T., Pietsch, M., Christiaens, D., Jeurissen, B., Yeh, C.-H., and Connelly, A. (2019). MRtrix3: A fast, flexible and open software framework for medical image processing and visualisation. *NeuroImage*, 202:116137.
